# Variation in Mesopic Retinal Sensitivity Relative to Distance from Geographic Atrophy in Age-Related Macular Degeneration

**DOI:** 10.1016/j.xops.2025.100879

**Published:** 2025-07-08

**Authors:** Souvick Mukherjee, Emily Vance, Leon von der Emde, Thilaka Arunachalam, Tharindu De Silva, Alisa T. Thavikulwat, Christine Orndahl, Caroline Nyaiburi, Maria Abraham, Keri Hammel, SriniVas R. Sadda, Emily Y. Chew, Maximilian Pfau, Wai T. Wong, Brett G. Jeffrey, Tiarnán D.L. Keenan

**Affiliations:** 1 National Eye Institute, National Institutes of Health, Bethesda, Maryland; 2 The Emmes Company, LLC, Rockville, Maryland; 3 Doheny Eye Institute, Pasadena, California; 4 Department of Ophthalmology, University of California Los Angeles, Los Angeles, California; 5 Department of Ophthalmology, University Hospital Basel, Basel, Switzerland; 6 Roche Pharmaceutical Research and Early Development, Roche Innovation Center Basel, Basel, Switzerland; 7 Tiresias Biopharma Consulting LLC, Half Moon Bay, California

**Keywords:** Geographic atrophy, Mesopic microperimetry, Outcome measures, Retinal sensitivity, Transitional zone

## Abstract

**Purpose:**

To analyze the relationship between distance from geographic atrophy (GA) lesion borders and mesopic retinal sensitivity in age-related macular degeneration (AMD).

**Design:**

Exploratory analyses of the longitudinal microperimetry dataset from a recent phase II, prospective, single-arm, nonrandomized trial of oral minocycline for GA progression.

**Participants:**

Individuals with GA from AMD in ≥1 eye.

**Methods:**

Mesopic retinal sensitivity was assessed longitudinally with a fundus-guided microperimetry device at baseline, month 3, and every 6 months thereafter, using a custom T-shaped test pattern centered on the fovea. Individual test loci were superimposed on an aligned fundus autofluorescence image and distance from the closest GA lesion border (GA distance) was computed. The relationship between GA distance and retinal sensitivity was analyzed in study eyes using repeated-measures regression.

**Main Outcome Measures:**

Mesopic retinal sensitivity.

**Results:**

The study population comprised 26 study eyes from 26 participants (mean age 74.2 years). Retinal sensitivity of extralesional testing loci increased steeply, as a quadratic function, between 0° and 2.05° (i.e., knot at 2.05°; 95% confidence interval [CI] 1.26°–2.84°) of GA distance. Beyond 2.05°, it increased linearly and less steeply. In nonlinear analyses accounting for nesting, a significant effect of GA distance on retinal sensitivity was observed. For GA distances <2.05°, sensitivity increased quadratically by approximately 1.99 decibels (dB)/° (95% CI: 1.15, 2.83 dB/°; *P* < 0.001) or higher. For GA distances ≥2.05°, sensitivity increased at 0.56 dB/° (95% CI: 0.42, 0.70 dB/°; *P* < 0.001). There was also a significant effect of time on sensitivity (estimate: −0.07 dB/month; 95% CI: −0.08, −0.06 dB/month; *P* < 0.001).

**Conclusions:**

The results demonstrate a perilesional zone 2° (∼580 μm) around the GA border in which retinal sensitivity changes steeply according to GA distance. This zone presents an important focus for closer evaluation in interventional studies examining potential efficacy for the preservation or recovery of retinal function. With GA progression, decreased retinal sensitivity expands ahead of GA expansion itself, as an advancing wave. Overall, the degree and extent of decreased visual function beyond GA borders have important implications for the design of clinical trials, decision-making in clinical practice, and insights into AMD pathophysiology.

**Financial Disclosure(s):**

Proprietary or commercial disclosure may be found in the Footnotes and Disclosures at the end of this article.

Geographic atrophy (GA), the nonexudative late-stage manifestation of age-related macular degeneration (AMD), is defined by the presence of complete retinal pigment epithelium and outer retinal atrophy with a diameter >250 μm.^1^ Foci of GA typically begin in the parafoveal region and expand gradually over time.^2^^,^^3^ When GA progresses to involve the fovea, with sufficient size, it is usually accompanied by severely decreased visual acuity.^2^^,^^4^, ^5^, ^6^, ^7^ Consequently, visual acuity may remain relatively stable for prolonged periods, such that acuity is not an ideal measure for tracking GA progression.^8^^,^^9^

Fundus-guided perimetry (or microperimetry), which uses eye-tracking to present test stimuli precisely to prespecified locations, provides a reliable method for measuring retinal sensitivity at specific retinal loci.^10^, ^11^, ^12^ The combination of fundus tracking and the “follow-up” function allows for monitoring of the same loci across multiple time points, making it a critical tool for longitudinal studies in diseases with unstable fixation. As a result, assessing macular sensitivity has become an essential outcome measure in clinical trials designed to slow vision loss in GA.^11^^,^^12^

Macular areas affected by GA typically exhibit dense scotomata in the corresponding visual field.^13^, ^14^, ^15^ However, our spatial understanding of visual function in macular regions outside GA remains incomplete, with few studies exploring this question.^15^, ^16^, ^17^, ^18^ Some authors propose a precipitous decline in visual function near GA borders, at the transitional zone.^16^ Others suggest a more gradual change.^17^^,^^18^ If the transition is indeed gradual, it remains unclear how far subnormal sensitivity may extend from GA lesions.^19^ This has important implications both for understanding AMD pathophysiology and for optimizing therapeutic strategies, including clinical trial design, interpretation, and decision-making in clinical practice.

We recently completed a phase II trial to evaluate the safety and potential efficacy of oral minocycline for GA in AMD.^20^ The trial involved the collection of longitudinal mesopic microperimetry data from all study eyes, following a prespecified protocol using a T-shaped testing pattern that extended 15° temporally, 12° superiorly, and 12° inferiorly from the fovea.^20^ As described previously, this pattern was designed to maximize useful information while minimizing participant burden, by halving the distance between loci and extending the pattern further into the peripheral macula, compared with the typical 10-2 circular grid pattern.^11^^,^^12^^,^^21^ These factors make the pattern particularly suited to mapping retinal sensitivity as a function of distance from GA borders.

In the phase II trial, no significant treatment effect in slowing GA progression was observed, either from the primary outcome measure of GA area or from exploratory analyses of the microperimetry data.^20^ This dataset provides an important opportunity to investigate changes in retinal sensitivity outside areas affected by GA. Specifically, the aims of this analysis were to examine changes in retinal sensitivity with increasing distance from GA, assess whether these changes are precipitous or gradual, and explore how far subnormal sensitivity extends from GA borders.

## Methods

The study design for the phase II trial evaluating minocycline for GA in AMD has been described previously.^20^ In brief, the study comprised a prospective, single-arm, phase II trial to evaluate the safety and possible efficacy of oral minocycline as a treatment to slow GA progression. The study was conducted at the National Institutes of Health (NIH) Clinical Center, Bethesda, Maryland, and the Bristol Eye Hospital, Bristol, United Kingdom. The protocol was approved by the NIH Institutional Review Board and the South Central-Oxford B Research Ethics Committee and adhered to the tenets of the Declaration of Helsinki. Written informed consent was obtained from each participant before enrollment. The study was registered at www.clinicaltrials.gov (NCT02564978; registration date, October 1, 2015). Study oversight was provided by an independent external Data and Safety Monitoring Committee that approved the protocol prior to trial initiation and reviewed study data approximately every 6 months. This manuscript relating to an exploratory outcome was not reviewed by the Data and Safety Monitoring Committee, according to the Data and Safety Monitoring Committee policy on review of manuscripts for exploratory outcomes.

The inclusion criteria have been described previously.^20^ Eligible participants were ≥55 years old and had GA secondary to AMD in 1 or both eyes. If both eyes of an individual participant met the eligibility criteria, the eye with better best-corrected visual acuity was chosen as the study eye. At the eye level, the study eye eligibility criteria included: (1) GA area >0.5 and <7.0 disc areas (approximately 1.27–17.81 mm^2^) on fundus autofluorescence (FAF) imaging; (2) best-corrected visual acuity ETDRS letter score ≥19 (Snellen 20/400); and (3) no current evidence or history of treatment for macular neovascularization. Eyes with or without GA foveal involvement were included.

Enrolled participants were followed during an initial 9-month run-in phase without the administration of minocycline, with in-clinic assessments at baseline and months 3, 6, and 9. At month 9, the participants began taking oral minocycline 100 mg twice daily until study termination, with in-clinic assessments at months 12, 15, and every 6 months thereafter. The primary outcome measure was assessed at month 33 (i.e., after 24 months on minocycline), and the study ended at month 45.

### Microperimetry Testing

**Figure 1:**
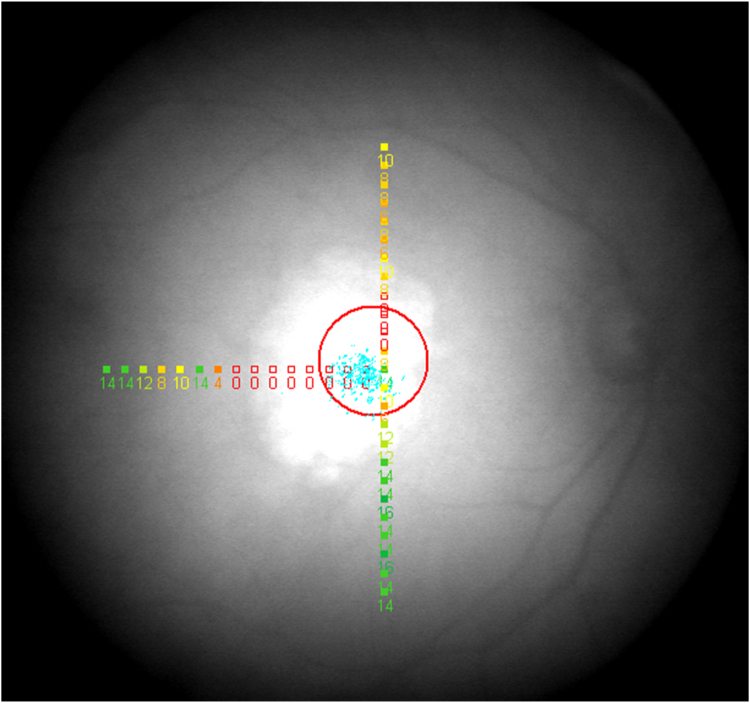
Mesopic microperimetry testing results in a study eye. The T-shaped testing grid consists of 40 evenly spaced testing loci at 1° apart, extending from the anatomic fovea (15° temporally, 12° superiorly, and 12° inferiorly). The values refer to the amount of attenuation (dB) relative to the maximum stimulus intensity of 127 cd/m^2^. For example, 10 dB corresponds to a sensitivity of 12.7 cd/m^2^. The loci and values are color-coded, ranging from red (0 dB) to green (20 dB). The empty red squares labeled zero indicate loci at which the participant did not respond to the maximum stimulus intensity (i.e., absolute scotoma). The red circle represents the fixation stimulus seen by the participant, while the blue dots show the results of fixation tracking (monitored at 25 Hz). dB = decibel.

The assessment of retinal sensitivity by mesopic microperimetry has been described previously.^21^^,^^22^ In brief, at baseline, month 3, and every 6 months thereafter, retinal sensitivity of the study eyes was assessed by mesopic microperimetry, using the MP-1 microperimeter (Nidek Technologies). For each study eye, prior to the first test, the anatomic fovea was identified using spectral-domain OCT (Spectralis HRA + OCT, Heidelberg Engineering Inc), and a horizontal line scan passing through the foveal center was uploaded into the MP-1 software. This allowed the test pattern to be centered on the fovea (Fig 1). After pupillary dilation, microperimetry testing was conducted under mesopic conditions in a darkened room, without prior dark adaptation. Testing was performed using a custom T-shaped pattern, consisting of 40 evenly spaced testing loci with their center-points 1° apart (Fig 1). The intersection of the T-shaped grid was placed at the anatomic fovea, with the 3 arms (or axes) of the T extending 15° temporally, 12° superiorly, and 12° inferiorly. A white Goldmann III stimulus (0.43° or 125 μm) was displayed for 200 ms at each testing locus. The stimulus intensities ranged from 127 to 1.27 cd/m^2^, corresponding to macular sensitivities of 0 to 20 decibels (dB). The starting stimulus light attenuation was set at 10 dB, and a 4-2 staircase strategy was used. A red circle with a 3° radius was set as the fixation target on a white background luminance of 4 apostilbs (1.27 cd/m^2^). After the first microperimetry test, all subsequent testing was performed in the instrument’s “follow-up mode” to ensure spatial alignment of individual testing loci over longitudinal testing during the study. If participant cooperation was unreliable or poor, the investigator had the discretion to stop testing according to the study protocol.

### FAF Image Acquisition and Grading for GA

Multimodal imaging was performed at all study visits following the prespecified protocol, as previously described.^20^^,^^22^ In brief, multimodal imaging comprised short-wavelength “blue” FAF, color fundus photography, and infrared reflectance or spectral-domain OCT. The FAF was performed as follows (Spectralis HRA2, Heidelberg Engineering Inc): wavelengths of 488 nm (excitation) and 500 nm (emission), captured in high-speed mode, with 15 averaged images, normalization on, and imaging field of 30 degrees (768 x 768 pixels). The FAF images were graded by an external reading center (Doheny Image Reading and Research Lab), by 2 independent graders masked to participant identifiers, to study visits, and to microperimetry data. The grading was performed according to a standardized protocol, and the total area of definitely decreased autofluorescence was annotated and quantified by planimetry.^23^^,^^24^

### Overlaying of Sensitivity Measurements on FAF Images and Calculation of Distances between Test Points and GA

**Figure 2:**
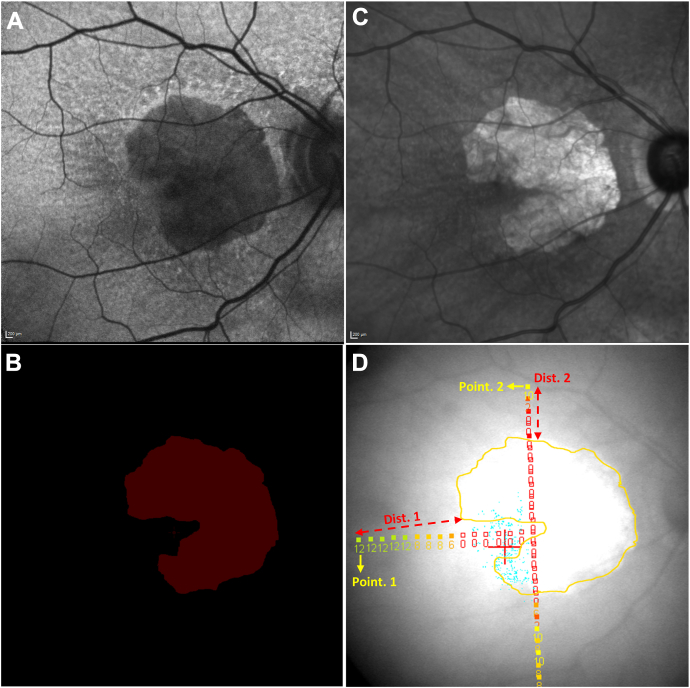
Overlaying of retinal sensitivity measurements on FAF images and calculation of distances between test points and GA: (**A**) FAF image; (**B**) GA annotation by reading center grading; (**C**) infrared reflectance image; (**D**) retinal sensitivity measurements overlaid on GA annotation. The overlay permitted calculation, for each microperimetry testing locus, of the distance between testing locus and nearest GA pixel, exemplified in (**D**) for 2 loci. Note that, in (**D**), “Dist. 1” is oblique because it represents the shortest distance between testing locus “Point. 1” and the nearest GA pixel. The loci and values are color-coded, ranging from red (0 dB) to green (20 dB). Dist. = distance; FAF = fundus autofluorescence; GA = geographic atrophy.

Retinal sensitivity measurements were overlaid on the corresponding FAF images, using methods described previously.^22^ To explore changes in retinal sensitivity according to increasing distance from GA, the Euclidean distance (i.e., shortest, straight-line distance) between each test point and the nearest pixel with GA was measured (hereafter, also referred to as GA distance; Fig 2). In the case of GA multifocality, for each test point, the Euclidean distance to each GA lesion was computed and the smallest distance was used in the analyses. This procedure was repeated for each time point in the longitudinal dataset. To account for GA expansion over time, the distances between the test points and the nearest GA pixels were calculated separately at each time point.

### Statistical Analysis

Given that previous longitudinal analyses of both structural and functional data (including the microperimetry data) were not consistent with statistically significant or clinically meaningful efficacy of oral minocycline in slowing GA progression,^20^^,^^21^ all microperimetry data (i.e., both before and after beginning minocycline dosing) were analyzed as a single pooled natural history study dataset. To decrease potential heterogeneity in data from differences in technician procedures during microperimetry testing at different sites, only data from the NIH site were used, while data from the Bristol Eye Hospital site (n = 7 participants) were excluded, in consistency with the approach used in recent analyses.^20^^,^^21^

First, to visualize the relationship between GA distance and retinal sensitivity, a scatter plot with locally estimated scatterplot smoothing (LOESS) was utilized, with a smoothing parameter of 0.5. Retinal sensitivity was plotted against distance from GA, using data from all 3 microperimetry axes (i.e., superior, temporal, and inferior testing axes), both using baseline data only and separately by visit.

Second, to analyze the relationship between GA distance and retinal sensitivity, a nonlinear mixed-model repeated-measures regression was performed, with retinal sensitivity as the outcome measure. Given the trends observed from the locally estimated scatterplot smoothing plots, a quadratic curve was fit for smaller distances from GA and a straight line was fit for larger distances from GA. The distance from GA where the relationship changed from a quadratic curve to a straight line was referred to as a knot, or change-point, and was estimated as a parameter from the model. The hierarchical nature of the data (i.e., nesting of loci within axis, within axis orientation [horizontal or vertical], within participant visit) was accounted for via a random effect. The final model is described in detail in the Material S1 (available at www.ophthalmologyscience.org). In supplementary analyses, the statistical modeling was repeated with inclusion of Euclidean distance between the test point and the foveal center point as an additional covariate. All statistical analyses were performed using PROC NLMIXED in SAS version 9.4 (SAS Institute).

## Results

### Baseline Demographics and Ocular Characteristics

The study population for these analyses has been described previously.^20^ Of the 30 participants from the NIH site, 4 were excluded owing to the imaging data being either missing or not captured in follow-up mode. Therefore, the analysis population comprised 26 participants. The baseline characteristics of the study population are shown in Table 1. The mean age was 74.2 years (standard deviation 7.5 years, range 63–89 years). The mean follow-up, based on microperimetry testing, was 26.5 months (standard deviation 13.1 months, range 3–51 months). The total number of microperimetry tests was 135, corresponding to a mean of 5.2 visits per participant (standard deviation 1.8 visits, range 1–8 visits). There were no instances where the GA did not intersect with the T-shaped testing pattern.

**Table 1:** Participant Demographic and Ocular Characteristics at Study Baseline

Variable	Characteristic or Statistic	Value
	N	26
Sex	Male, N (%)	11 (42)
	Female, N (%)	15 (58)
Age	Mean (SD; range); years	74.2 (7.5; 63–89)
Ethnicity	Hispanic or Latino, N (%)	1 (4)
	Not Hispanic or Latino, N (%)	24 (92)
	Unknown, N (%)	1 (4)
Race	Asian, N (%)	1 (4)
	Black, N (%)	1 (4)
	White, N (%)	24 (92)
Fovea involving GA based on FAF (study eye)	Subfoveal, N (%)	13 (50)
	Not subfoveal, N (%)	13 (50)
Square root of GA area	Mean (SD; range); mm	2.6 (1.0; 1.0–4.9)
BCVA letter score; Snellen distance∗	Mean (SD; range)	70.8 (16.5; 27–90); 59.1 (73.0; 16–320)
LLVA letter score; Snellen distance∗	Mean (SD; range)	53.4 (16.1; 23–77); 120.1 (105.7; 32–400)

BCVA = best-corrected visual acuity; FAF = fundus autofluorescence; GA = geographic atrophy; LLVA = low-luminance visual acuity; SD = standard deviation.

Percentages are rounded to the nearest whole number.

∗ Snellen distance represents the denominator in the 20/X Snellen notation, that is, the size of letters read on a Snellen visual acuity chart at 20 feet; for <20/800 Snellen values, 800 was used as the Snellen distance.

**Figure 3:**
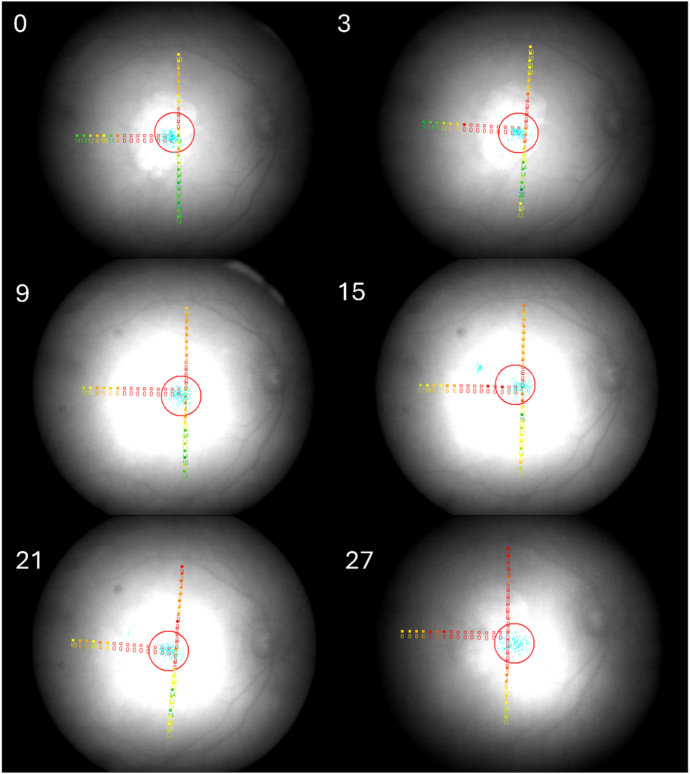
Longitudinal microperimetry data from 1 study eye during the study period. Sensitivity values are presented in dB, with absolute scotomas indicated by empty red squares, and fixation results shown as blue dots within a red circle. The study month appears in the upper-left corner. Note that, while small turns of the eye or head can skew the retinal image, the T-shaped testing pattern always overlays the same retinal areas. The loci and values are color-coded, ranging from red (0 dB) to green (20 dB). dB = decibel.

The raw microperimetry data for 1 participant during longitudinal follow-up are shown in Figure 3.

### Plots of Mean Retinal Sensitivity against Distance from GA

**Figure 4:**
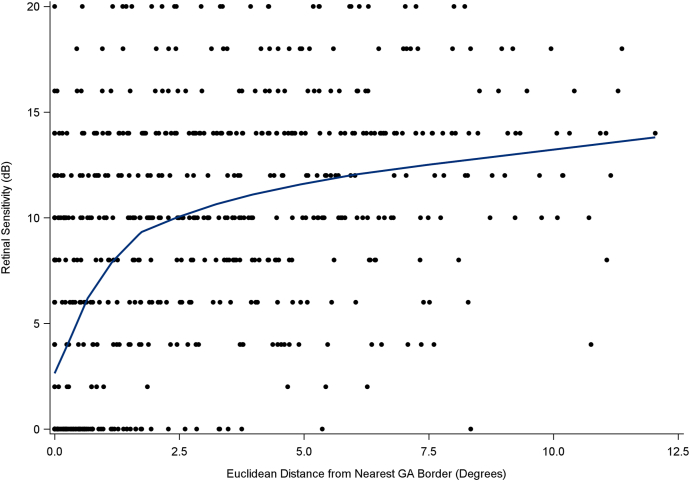
Plot of retinal sensitivity against distance from GA, based on data from the baseline time point. Locally estimated scatterplot smoothing was performed with a smoothing parameter of 0.5. dB = decibel; GA = geographic atrophy.

The plot of retinal sensitivity against distance from GA, including the locally estimated scatterplot smoothing curve fit, using data from the baseline time point only, is shown in Figure 4. The relationship appeared to change with increasing distance from GA; retinal sensitivity (expressed in decibels) increased more steeply at lower distances, followed by a less steep, linear increase at higher distances.

Similar plots using data from the baseline time point only, but with each microperimetry testing axis considered separately, are shown in Figure S5 (available at www.ophthalmologyscience.org). A similar pattern was observed in each case, though retinal sensitivity was generally lower for the superior axis. The position of the knot appeared relatively consistent between the different axes.

**Figure 6:**
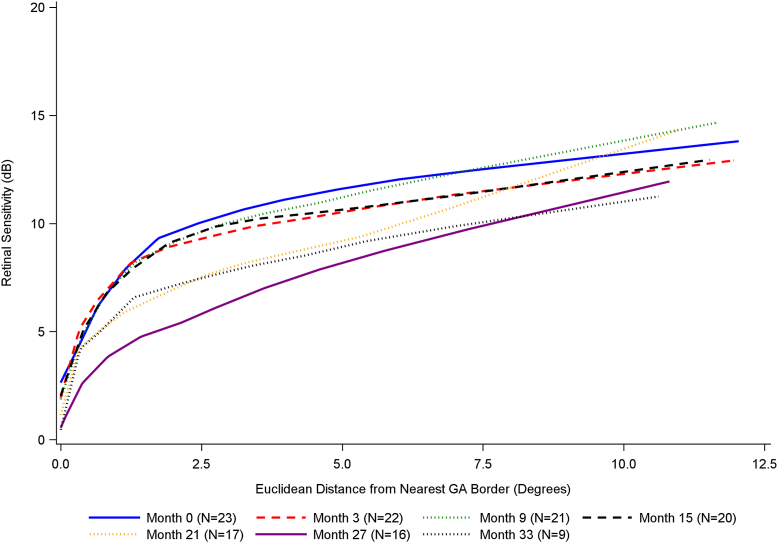
Plot of LOESS (smoothing parameter = 0.5) curves of retinal sensitivity against distance from GA, with curves for each time point shown separately. N corresponds to the number of participants with available data. Time points after month 33 are not included, owing to low participant numbers, though these data were included in the statistical modeling. dB = decibel; GA = geographic atrophy; LOESS = locally estimated scatterplot smoothing.

Locally estimated scatterplot smoothing curves of retinal sensitivity against distance from GA, with data shown separately for each time point in the longitudinal dataset, are shown in Figure 6 and Figure S7 (available at www.ophthalmologyscience.org). Figure 6 includes curves for time points up to month 33, whereas Figure S7 includes curves for all time points, even the time points after month 33, where fewer data were available, as all time points were used in the statistical model. In general, for most time points, the pattern of change was relatively similar to that of the baseline data. The position of the knot appeared relatively consistent between the different time points. The curve for month 39 deviated slightly for larger distances, likely due to the small amount of data available (i.e., 2 participants).

### Statistical Analysis

The results of the statistical analyses are shown in Table 2. The knot, that is, the GA distance where the relationship between GA distance and retinal sensitivity changed from quadratic to linear, was estimated at a GA distance of 2.05° (95% confidence interval [CI]: 1.26°, 2.84°). For GA distances <2.05°, there was a 1.53 + 0.93∗(2.05 – GA distance) dB change in retinal sensitivity for every 1° increase in GA distance, holding time constant. Examples of the relationship between GA distance and retinal sensitivity, holding time constant, for GA distances <2.05°, were as follows:

• Retinal sensitivity increased by 2.96 dB (95% CI = 2.46, 3.46 dB; *P* < 0.001) when the GA distance increased from 0° to 1°, with 0.5° corresponding to the midpoint of this increase.
• Retinal sensitivity increased by 2.50 dB (95% CI = 2.13, 2.87 dB; *P* < 0.001) when the GA distance increased from 0.5° to 1.5°, with 1° corresponding to the midpoint of this increase.
• Retinal sensitivity increased by 2.04 dB (95% CI = 1.24, 2.83 dB; *P* < 0.001) when the GA distance increased from 1° to 2°, with 1.5° corresponding to the midpoint of this increase.

**Table 2:** Results of Nonlinear Mixed-Model Regression Models Assessing the Relationship between Retinal Sensitivity and Distance from GA

Parameter	Estimate (95% CI)	*P* Value
Time	−0.07 (−0.08, −0.06)	<0.001
Before the knot (GA distance <2.05°∗)		
GA distance	−1.53 (−3.48, 0.43)	0.125
Quadratic GA distance	−0.46 (−1.01, 0.09)	0.100
After the knot (GA distance ≥2.05°∗)		
GA distance	0.56 (0.42, 0.70)	<0.001

CI = confidence interval, GA = geographic atrophy.

Measurement units: retinal sensitivity = decibel, GA distance = degrees, quadratic GA distance = degrees^2^, time = months.

∗ 95% CI of the knot of 2.05 = (1.26, 2.84).

For GA distances ≥2.05°, retinal sensitivity increased by 0.56 dB (95% CI = 0.42, 0.70 dB; *P* < 0.001) for every 1° increase in GA distance, holding time constant.

**Figure 8:**
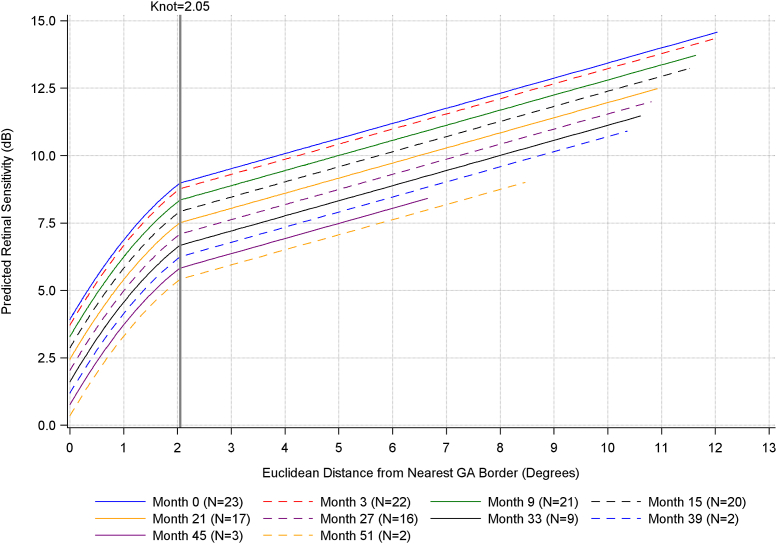
Plot of predicted retinal sensitivity calculated using PROC NLMIXED. N corresponds to the number of participants with available data. dB = decibel; GA = geographic atrophy.

Predicted retinal sensitivity for varying GA distances, based on the model, is presented by visit in Figure 8.

The results of supplementary analyses, in which the statistical analysis was repeated with inclusion of foveal distance as an additional covariate, are shown in Table S3 (available at www.ophthalmologyscience.org). The estimate associated with foveal distance was nonsignificant and small in magnitude, and its inclusion in the model did not materially alter the other estimates from the model or overall model fit.

## Discussion

This study examined the relationship between distance from GA and retinal sensitivity, using mesopic microperimetry data obtained in a clinical trial setting. As expected, the findings demonstrate that retinal sensitivity increases with increasing distance from GA borders. Importantly, the relationship was concave down with steeper increases in retinal sensitivity up until a GA distance of approximately 2° (∼580 μm) but was linear and less steep for GA distances >2°. Hence, the data support both the idea of a very steep, if not quite precipitous,^16^ change in visual function close to GA borders, as well as the idea of a gradual change,^17^^,^^18^ though only at greater distances from GA borders. Indeed, the data show that retinal sensitivity continues to increase gradually (and linearly) even at relatively high distances from GA (e.g., 7°–10°, or 2030–2900 μm). Overall, the findings highlight that substantial impairment in visual function extends a long distance from GA borders themselves. This has important implications both for the design of interventional trials, for decision-making in clinical practice, and for understanding the sequence of events in AMD pathophysiology.

Interestingly, even the highest mean retinal sensitivity levels observed at the greatest distances from the GA border were still below the values expected at these macular locations (i.e., for individuals of similar ages without retinal disease, based on the same microperimetry device), of approximately 18 dB.^25^ This deficit of several decibels is similar to the difference observed in a previous cross-sectional study.^17^ Hence, the depressed retinal sensitivity values surrounding GA lesions appear to represent the combination of global macular sensitivity losses attributable to AMD and additional losses related specifically to GA, with the latter being highly spatially determined according to distance from the GA border.

Importantly, distance from GA was recalculated at each time point for every testing locus, to take into account progressive GA expansion over time. Despite this, evidence of a significant effect of time was observed, with mean retinal sensitivity decreasing over time at a given locus (i.e., in addition to the decrease attributable to the GA border having moved closer over time). This means that, during study follow-up, decreased retinal sensitivity expanded ahead of GA expansion itself, as an advancing wave—but that additional retinal sensitivity losses occurred gradually over time. The reasons for this are unclear but might be related to increased macular pathology with advancing age, such as increased incidence or extent of reticular pseudodrusen (which have been associated with outer retinal degeneration even in the absence of GA^26^).

In terms of disease biology, the zone bordering GA is a critical transition area. Histological analyses of human macular tissue have demonstrated a considerable transition zone in most eyes with GA, extending approximately 500 μm (and potentially as far as 1400 μm) from the GA border.^27^^,^^28^ This junctional zone is characterized by variable loss of photoreceptors (specifically the absence of rods and the presence of cones with no outer segments and few inner segments). Our findings indicate that the zone of decreased mesopic visual function extends beyond the GA junctional zone of 500 μm (∼1.7°) defined by retinal pigment epithelium cell abnormalities. Various structural biomarkers have been proposed to characterize relative scotomas in the outer nonlesional zone. One marker is FAF abnormalities, particularly increased FAF in the junctional zone, which has been associated with reduced retinal sensitivity.^18^ More recently, OCT-based photoreceptor integrity markers, such as outer nuclear layer thickness, ellipsoid zone thickness and reflectivity, external limiting membrane descent, outer retinal tubulations, and interdigitation zone discernibility, are increasingly used to assess functional impairment.^19^^,^^29^, ^30^, ^31^, ^32^, ^33^ Recent advances in artificial intelligence have enabled structure-function correlation models that integrate OCT and FAF-based features, allowing prediction of retinal dysfunction with a mean absolute error of 4.6 dB.^19^ Furthermore, emerging evidence suggests that OCT angiography-based measurements of choriocapillaris perfusion in the GA border zone correlate with retinal dysfunction, providing another potential biomarker of disease progression.^34^ To optimize structure-function assessments in GA, further comparative studies are needed to determine the most reliable structural biomarkers for predicting the extent of retinal dysfunction in the border zone.

In a previous cross-sectional study of 25 eyes of 25 participants with GA, by Pfau et al,^17^ mesopic microperimetry data were related to distance from GA. In agreement with the current study, this previous study also observed a gradual increase in sensitivity values on increasing distance from GA, with similar results at relatively small distances. However, the previous study observed a plateau without further apparent change in sensitivity at a relatively close distance from GA (approximately 2°, or 580 μm). The main difference between the 2 studies is the microperimetry grid design (in addition to differences in the microperimetry device and the cross-sectional vs. longitudinal acquisition of data). The previous study used a “patient-tailored” grid that was customized to the individual study eye by positioning test points along isocontour lines at 5 specific distances from GA, with a maximum distance of 3.0° (or 870 μm) from GA. As a result, the previous study was highly optimized toward the sensitivity slope within the immediate junctional zone to track atrophy progression over time. By contrast, the T-shaped grid used in the current study offers a balanced approach toward assessing the junctional zone and more peripheral sensitivity, providing additional data for large distances from GA borders.

Of note, both studies may appear to contrast with findings from a cross-sectional study of 36 eyes with GA, which reported a precipitous drop in sensitivity.^16^ In the previous study, sensitivity at the GA border itself was substantially decreased, compared with regions even a short (e.g., 250 μm) distance away, while sensitivity within the 500 μm junctional zone was similar to that of regions outside the junctional zone. Importantly, however, the GA contours in the previous study were defined by OCT rather than FAF. This may have led to different definitions of the GA transitional zone for the reasons discussed above.

These findings for visual function are consistent with previous reports of abnormalities in retinal structure beyond GA lesions.^19^^,^^35^^,^^36^ Specifically, recent studies have observed decreased photoreceptor layer thicknesses beyond GA borders; these were found to expand in advance of GA expansion itself. Of note, the degree of photoreceptor degeneration outside of GA varied widely and was observed to be associated with future GA progression rate and with altered treatment effect of local C3 inhibition.^19^^,^^35^^,^^36^ Similar associations might pertain to the degree of functional deficit outside GA. Our data are highly relevant for future clinical trials, as the T-shaped microperimetry grid used here allows for the prespecification of visual function end points that simultaneously capture therapeutic effects for both (1) GA progression and (2) macula-wide disease progression. For GA progression, sensitivity changes within 2° of baseline GA can serve as a key end point, while sensitivity changes beyond 4° from baseline GA could provide insight into macula-wide disease progression. The latter end point is particularly valuable in guiding strategic decisions on advancing potential therapies to earlier disease stages, namely intermediate AMD.

### Strengths and Limitations

This study has several strengths, including its prospective clinical trial setting, with microperimetry testing performed at regular intervals using a standardized protocol, with relatively long follow-up time. The combination of multimodal imaging at each visit and reading center grading of GA contours from FAF images enabled accurate ascertainment of distance to GA for each test locus at each time point. The statistical method was able to account for the hierarchical and longitudinal nature of the data and was able to fit different curves to different segments of the data, with the segmentation being determined by the model. However, the study has several potential limitations. The dataset came from an interventional clinical trial rather than a natural history study. However, previous analyses of both the microperimetry data itself, as well as structural outcome measures, have demonstrated no significant effect of the study drug.^20^^,^^21^ Other potential limitations include the exclusion of data from a small number of participants and the absence of data on reticular pseudodrusen presence or contours. Using a full grid would have increased sampling, potentially increasing accuracy of the modeling, but a full grid with large size and close interlocus spacing would be highly burdensome for participants. In cases of multifocal GA, the analyses of GA distance considered only the closest GA pixel, so the potential effects of multiple GA lesions at relatively similar distances to a test locus have not been evaluated. Finally, the study was not designed or powered for these analyses, so these analyses should be considered exploratory, and replication in other studies from different centers is warranted to validate these findings. Future studies, particularly from datasets with small and closely spaced test points, may help address whether similar rates of change in sensitivity may continue inward from GA margins (i.e., within GA lesions).

## Conclusions

This study of mesopic microperimetry data demonstrates that retinal sensitivity increases with increasing distance from GA, with a curvilinear relationship characterized by 2 parts: the increase is quadratic (concave down) within 2° (∼580 μm) but linear and less steep at greater distances. This supports the idea of both a very steep, if not quite precipitous, change in visual function at the transitional zone, as well as a gradual change at greater distances from GA borders. Overall, we find that a substantial degree of impairment in visual function extends a long distance beyond GA borders. This has important implications for the design of clinical trials, for decision-making in clinical practice, and for insights into AMD pathophysiology.
