# Supplementary material for: Variation in Mesopic Retinal Sensitivity Relative to Distance from Geographic Atrophy in Age-Related Macular Degeneration
Source: Ophthalmol Sci. 2025 Jul 8;5(6):100879. doi: 10.1016/j.xops.2025.100879 (PMC12362116; doi:10.1016/j.xops.2025.100879)
Supplement: Table S3 [file mmc1.pdf]

Supplementary Table 3. Results of non-linear mixed-model regression models assessing the relationship between retinal sensitivity and distance from geographic atrophy, with the inclusion of distance from fovea as a covariate.

**Parameter**	**Estimate (95% CI)**	**p-value**
Foveal Distance	0.06 (-0.01, 0.12)	0.110
Time	-0.07 (-0.08, -0.06)	<0.001
**Before the Knot (GA Distance < 2.05[a])**		
GA Distance	-1.51 (-3.43, 0.40)	0.120
Quadratic GA Distance	-0.45 (-1.00, 0.10)	0.106
**After the Knot (GA Distance >= 2.05[a])**		
GA Distance	0.52 (0.37, 0.67)	<0.001

Abbreviations: CI = confidence interval, GA = geographic atrophy.
Measurement units: retinal sensitivity = decibel, GA distance and foveal distance = degrees, quadratic GA distance = degrees^2, time = months
[a]95% CI of the knot of 2.05 = (1.29, 2.81).
