# Supplementary material for: Variation in Mesopic Retinal Sensitivity Relative to Distance from Geographic Atrophy in Age-Related Macular Degeneration
Source: Ophthalmol Sci. 2025 Jul 8;5(6):100879. doi: 10.1016/j.xops.2025.100879 (PMC12362116; doi:10.1016/j.xops.2025.100879)
Supplement: Figure S5 [file mmc2.pdf]

Supplementary Figure 5. Plot of Retinal Sensitivity Against Distance from Geographic Atrophy, Based on Data from the Baseline Time-Point, Separately for Each Microperimetry Testing Axis.

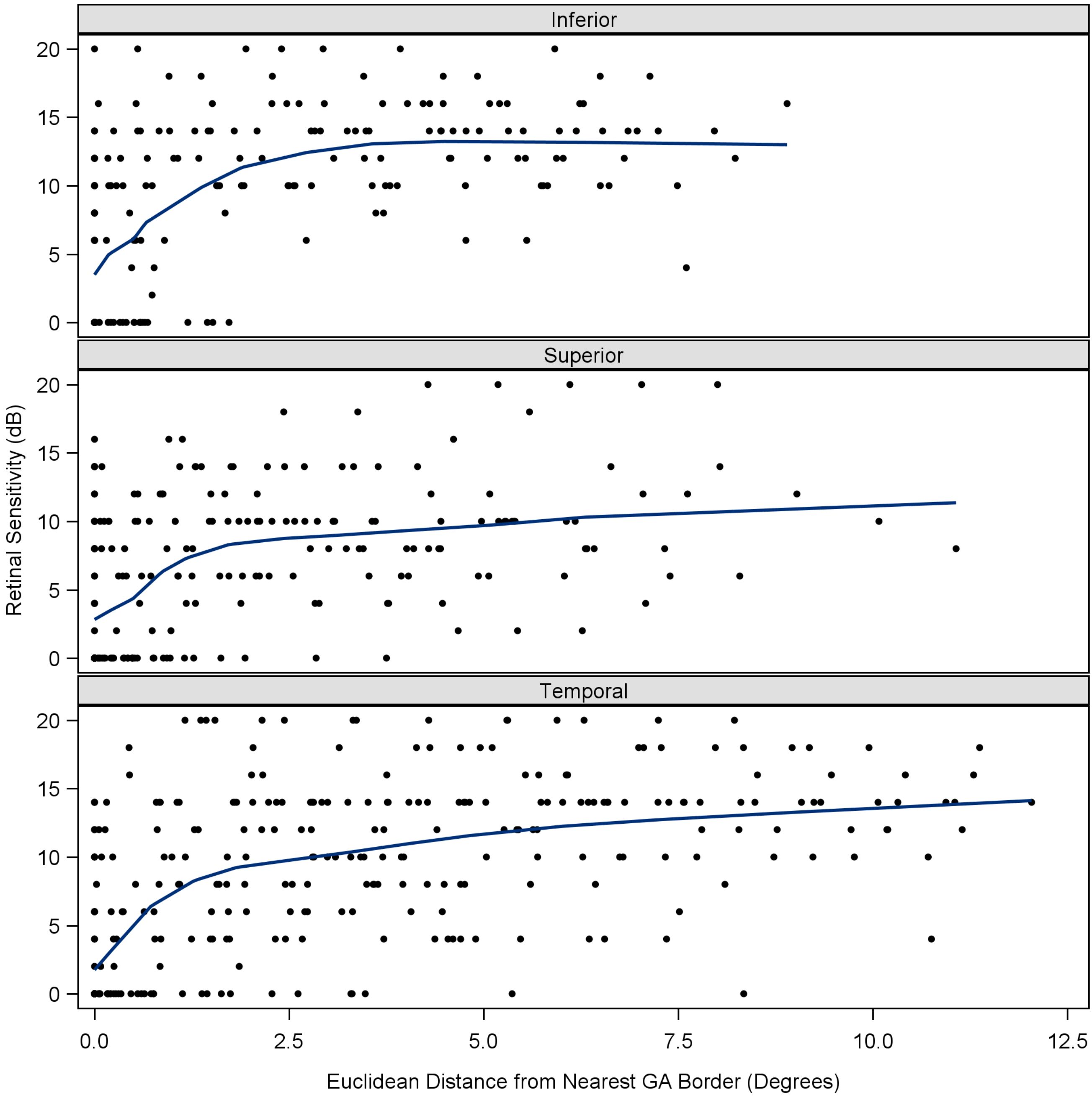

Locally estimated scatterplot smoothing (LOESS) was performed with a smoothing parameter of 0.5.
