# Supplementary material for: Variation in Mesopic Retinal Sensitivity Relative to Distance from Geographic Atrophy in Age-Related Macular Degeneration
Source: Ophthalmol Sci. 2025 Jul 8;5(6):100879. doi: 10.1016/j.xops.2025.100879 (PMC12362116; doi:10.1016/j.xops.2025.100879)
Supplement: Figure S7 [file mmc3.pdf]

Supplementary Figure 7. Plot of Locally Estimated Scatterplot Smoothing (LOESS) Curves of Retinal Sensitivity against Distance from Geographic Atrophy, with Curves for Each Time-Point Shown Separately.

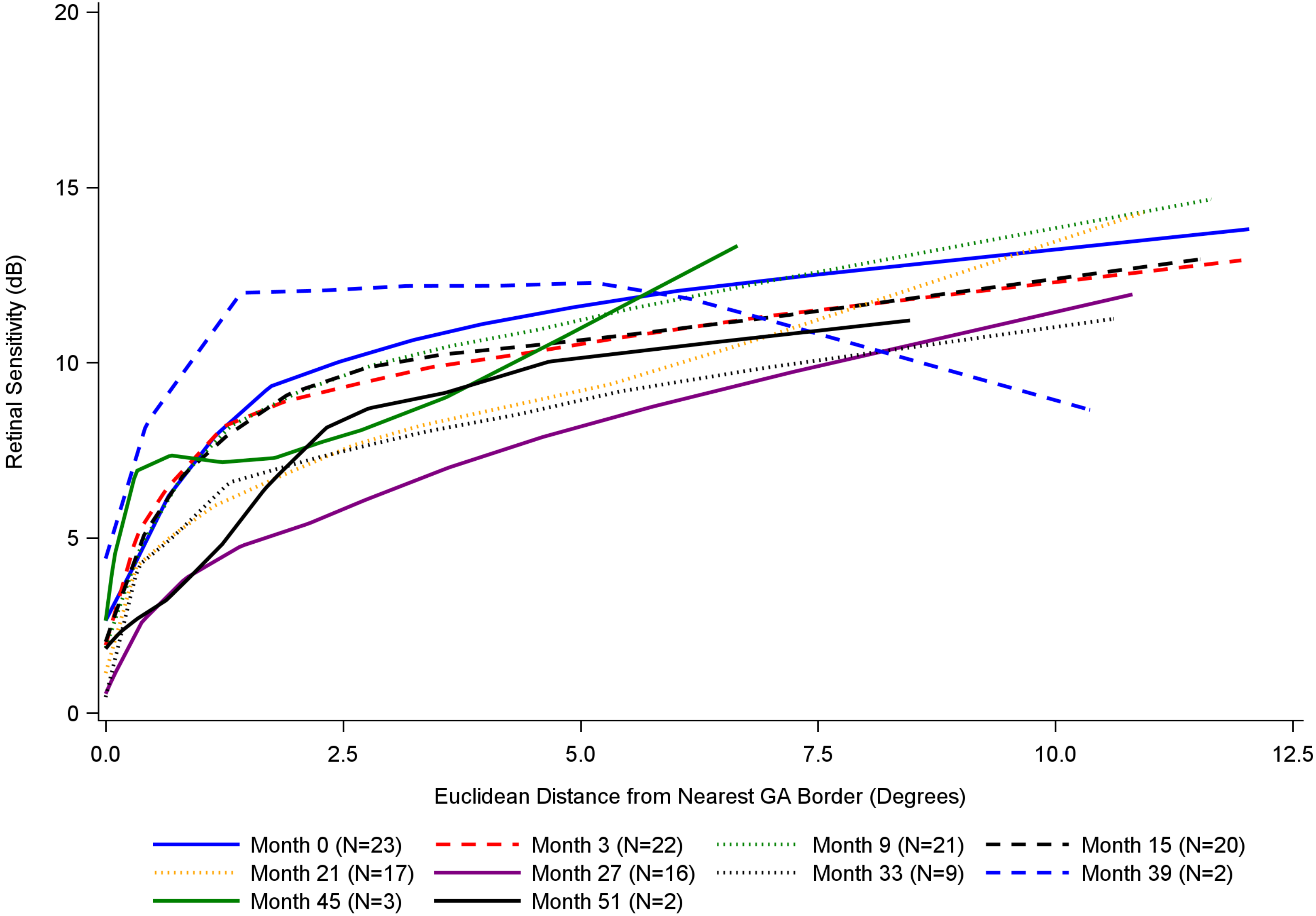

LOESS was performed with a smoothing parameter of 0.5.
N corresponds to the number of participants with available data.
