# Supplementary material for: Variation in Mesopic Retinal Sensitivity Relative to Distance from Geographic Atrophy in Age-Related Macular Degeneration
Source: Ophthalmol Sci. 2025 Jul 8;5(6):100879. doi: 10.1016/j.xops.2025.100879 (PMC12362116; doi:10.1016/j.xops.2025.100879)
Supplement: Material S1 [file mmc4.pdf]

Supplementary Material

The final statistical model was as follows:

$$\hat{Y}_{ijkt} = \begin{cases} \hat{\beta}_0 + \hat{\beta}_1 * (\widehat{knot} - GA\ Distance_{ijkt}) + \hat{\beta}_2 * (\widehat{knot} - GA\ Distance_{ijkt})^2 + \\ \hat{\beta}_3 * Time_{ijkt} + b_{0ijkt} + \varepsilon_{ijkt}, \\ GA\ Distance_{ijkt} < \widehat{knot} \\ \hat{\beta}_0 + \hat{\beta}_4 * (GA\ Distance_{ijkt} - \widehat{knot}) + \hat{\beta}_3 * Time_{ijkt} + \\ b_{0ijkt} + \varepsilon_{ijkt}, \\ GA\ Distance_{ijkt} \geq \widehat{knot} \end{cases}$$

Where:

$\hat{\boldsymbol{Y}}_{\boldsymbol{ijkt}}$ is the estimate of the mean retinal sensitivity for the ith loci on the jth axis in the kth scan orientation given the loci's geographic atrophy (GA) distance (in degrees) at time t

$\widehat{\mathbf{Knot}}$ is the estimated knot, i.e., the GA distance (in degrees) where the relationship between GA distance and retinal sensitivity changed from quadratic to linear

GA Distance$_{\mathbf{ijkt}}$ is the distance from the nearest GA pixel (in degrees) of the ith loci on the jth axis in the kth scan orientation at time t

Time$_{\mathbf{ijkt}}$ is the time in months from baseline for the ith loci on the jth axis in the kth scan orientation at time t

$\hat{\boldsymbol{\beta}}_{\mathbf{0}}$ is the estimate of the mean retinal sensitivity at baseline (i.e., time=0) when GA distance (in degrees) is equal to zero (i.e., when the loci is located within the GA)

$-\hat{\boldsymbol{\beta}}_{\mathbf{1}} - [\mathbf{2} * \hat{\boldsymbol{\beta}}_{\mathbf{2}}(\widehat{\mathbf{Knot}} - \mathbf{GA\ Distance_{ijkt}})]$ is the change in mean retinal sensitivity for every one degree increase in GA distance for GA distances less than the estimated knot (i.e., $\widehat{\mathbf{Knot}}$), holding time constant

$\hat{\boldsymbol{\beta}}_{\mathbf{4}}$ is the change in mean retinal sensitivity for every one degree increase in GA distance for GA distances greater than or equal to the estimated knot (i.e., $\widehat{\mathbf{Knot}}$), holding time constant

$\hat{\boldsymbol{\beta}}_{\mathbf{3}}$ is the change in mean retinal sensitivity for every one month increase in time from baseline, holding GA distance constant
